# Supplementary material for: Reduced Glycolysis and Cytotoxicity in Staphylococcus aureus Isolates from Chronic Rhinosinusitis as Strategies for Host Adaptation
Source: Int J Mol Sci. 2024 Feb 13;25(4):2229. doi: 10.3390/ijms25042229 (PMC10888669; doi:10.3390/ijms25042229)
Supplement: Supplementary file 1 [file ijms-25-02229-s001.zip › ijms-2860824-supplementary.pptx]

## Slide 1
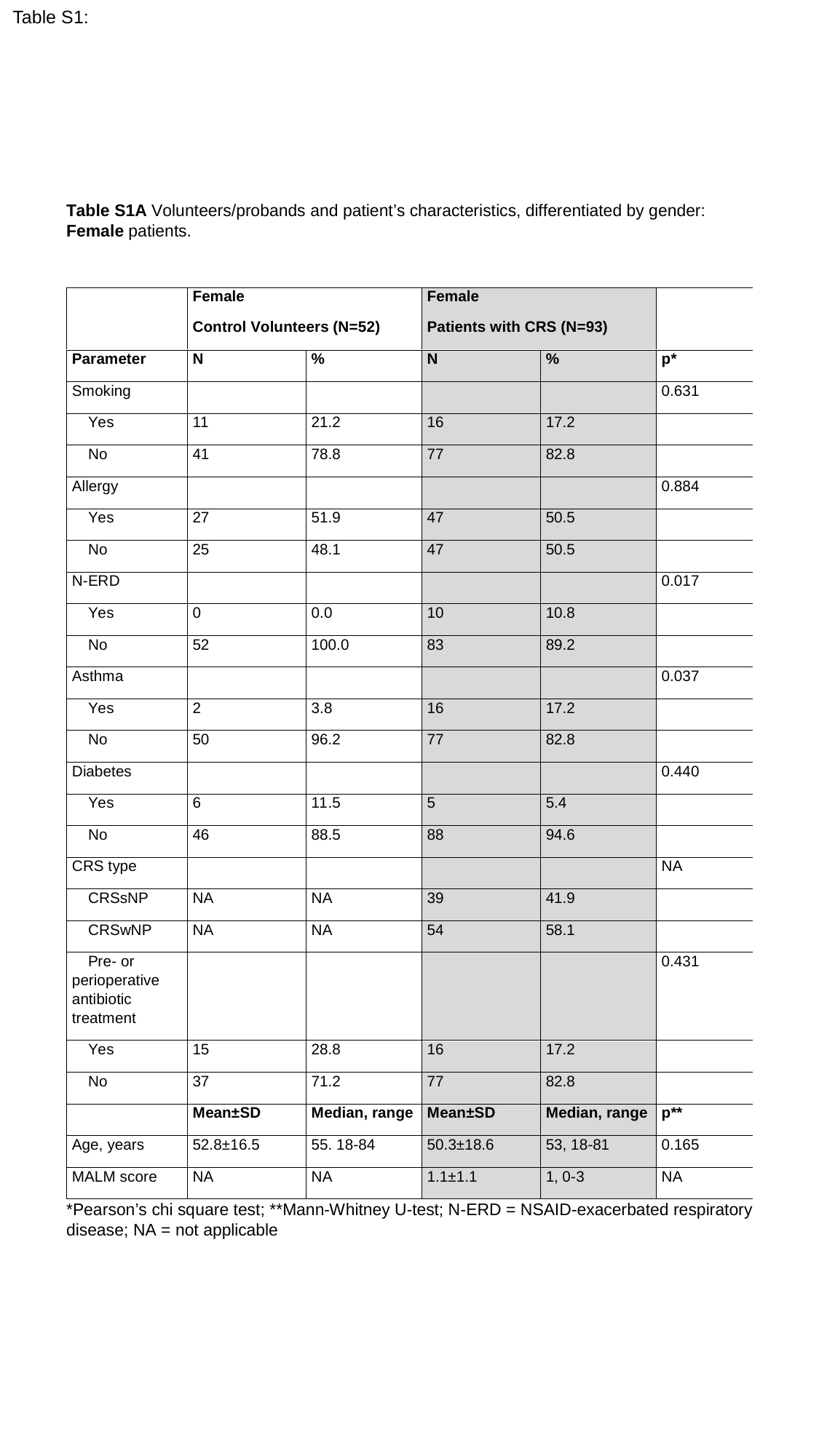

Table S1:

## Slide 2
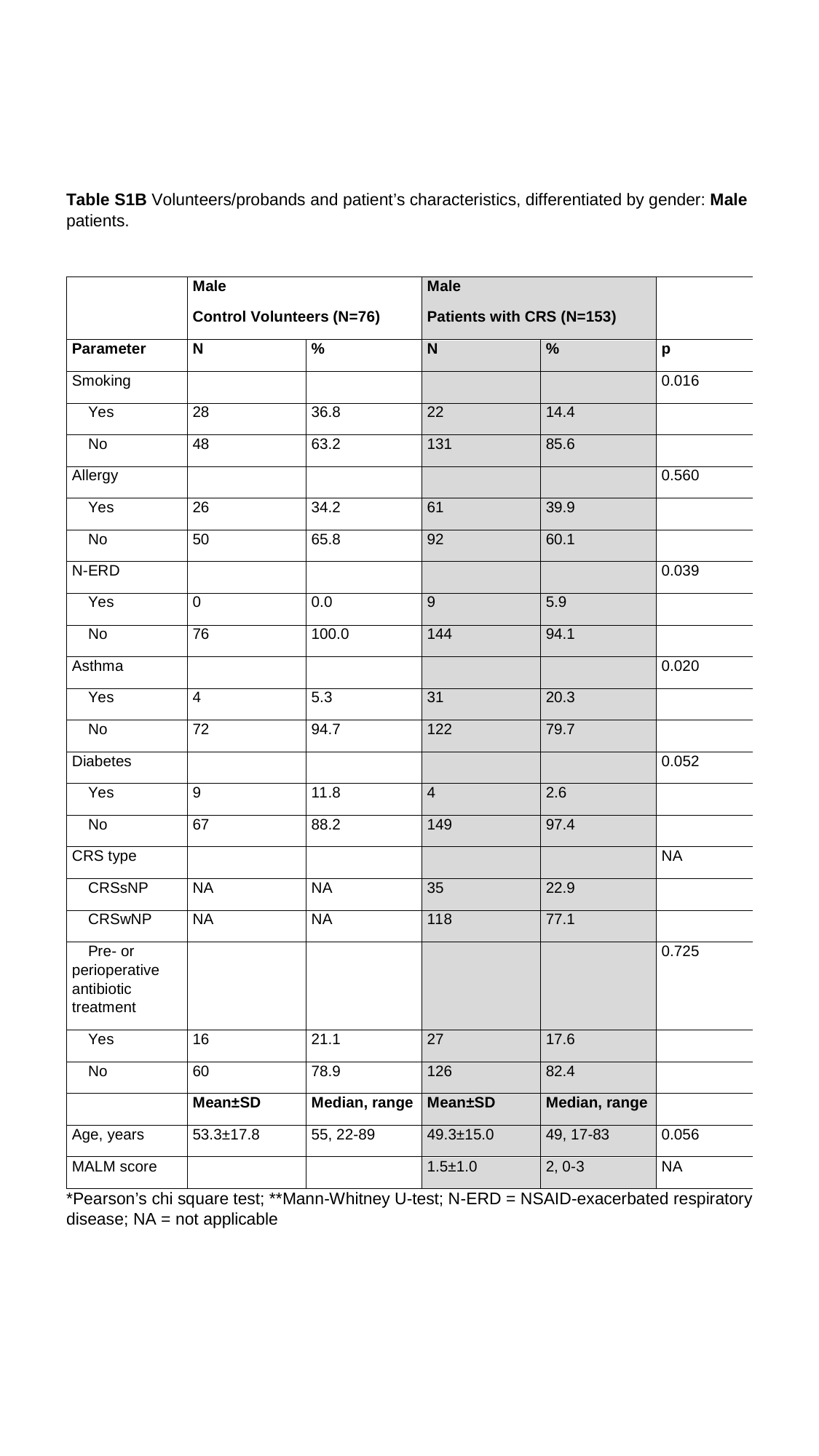

## Slide 3
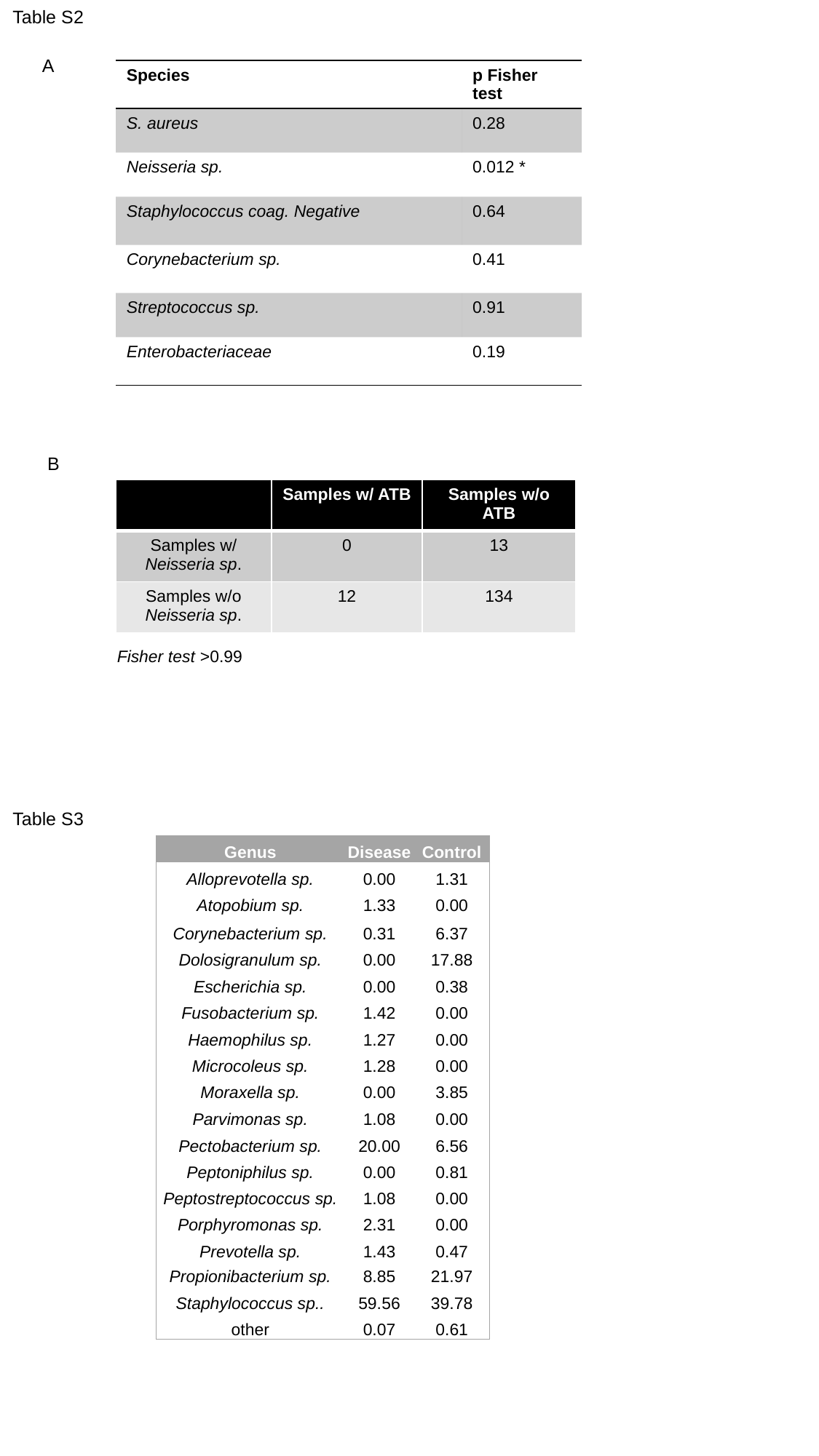

Table S2
A
| Species | p Fisher test |
| --- | --- |
| S. aureus | 0.28 |
| Neisseria sp. | 0.012 \* |
| Staphylococcus coag. Negative | 0.64 |
| Corynebacterium sp. | 0.41 |
| Streptococcus sp. | 0.91 |
| Enterobacteriaceae | 0.19 |
B
| | Samples w/ ATB | Samples w/o ATB |
| --- | --- | --- |
| Samples w/ Neisseria sp. | 0 | 13 |
| Samples w/o Neisseria sp. | 12 | 134 |
Fisher test >0.99
Table S3
| Genus | Disease | Control |
| --- | --- | --- |
| Alloprevotella sp. | 0.00 | 1.31 |
| Atopobium sp. | 1.33 | 0.00 |
| Corynebacterium sp. | 0.31 | 6.37 |
| Dolosigranulum sp. | 0.00 | 17.88 |
| Escherichia sp. | 0.00 | 0.38 |
| Fusobacterium sp. | 1.42 | 0.00 |
| Haemophilus sp. | 1.27 | 0.00 |
| Microcoleus sp. | 1.28 | 0.00 |
| Moraxella sp. | 0.00 | 3.85 |
| Parvimonas sp. | 1.08 | 0.00 |
| Pectobacterium sp. | 20.00 | 6.56 |
| Peptoniphilus sp. | 0.00 | 0.81 |
| Peptostreptococcus sp. | 1.08 | 0.00 |
| Porphyromonas sp. | 2.31 | 0.00 |
| Prevotella sp. | 1.43 | 0.47 |
| Propionibacterium sp. | 8.85 | 21.97 |
| Staphylococcus sp.. | 59.56 | 39.78 |
| other | 0.07 | 0.61 |

## Slide 4
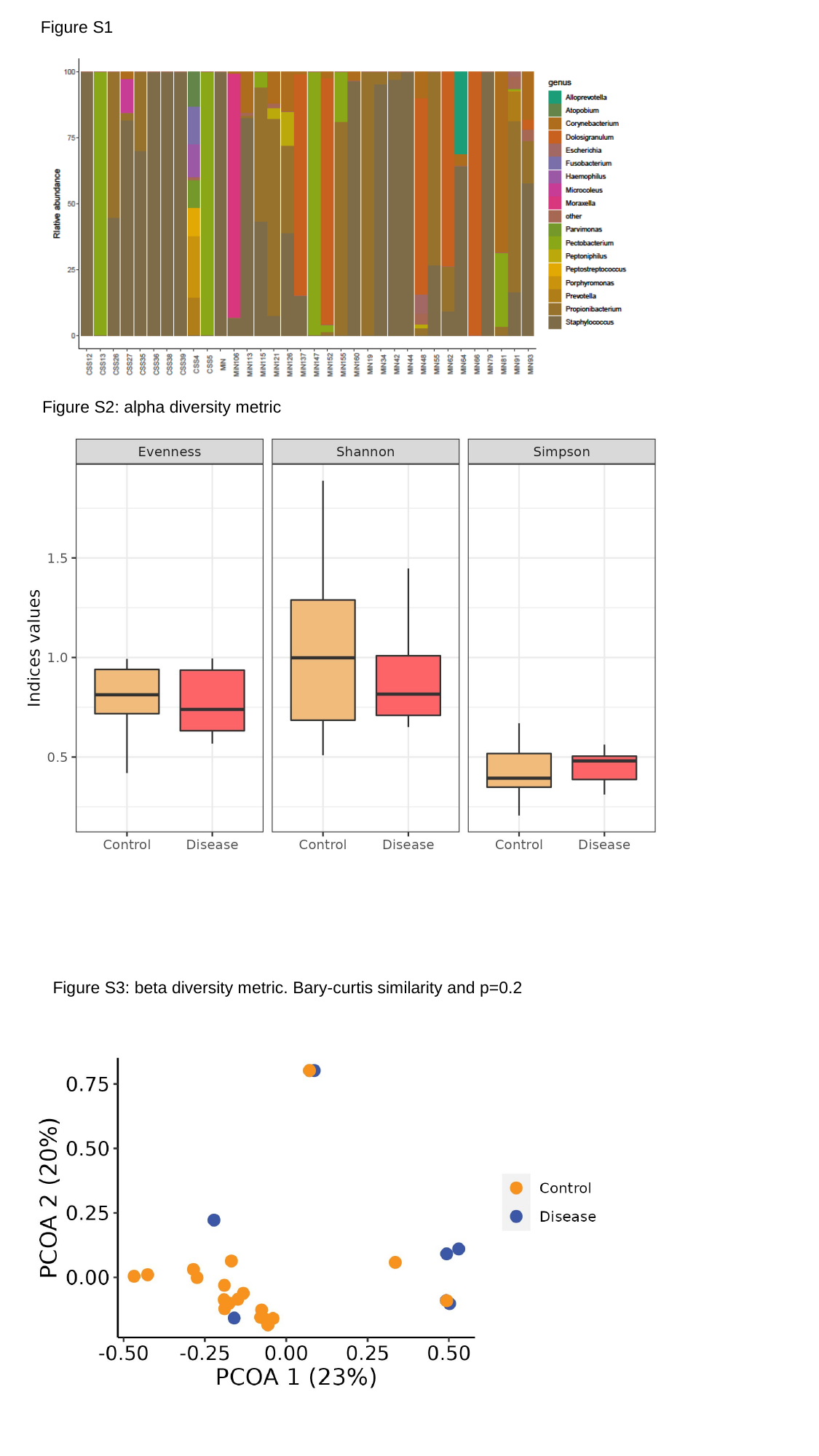

Figure S1
Figure S2: alpha diversity metric
Figure S3: beta diversity metric. Bary-curtis similarity and p=0.2

## Slide 5
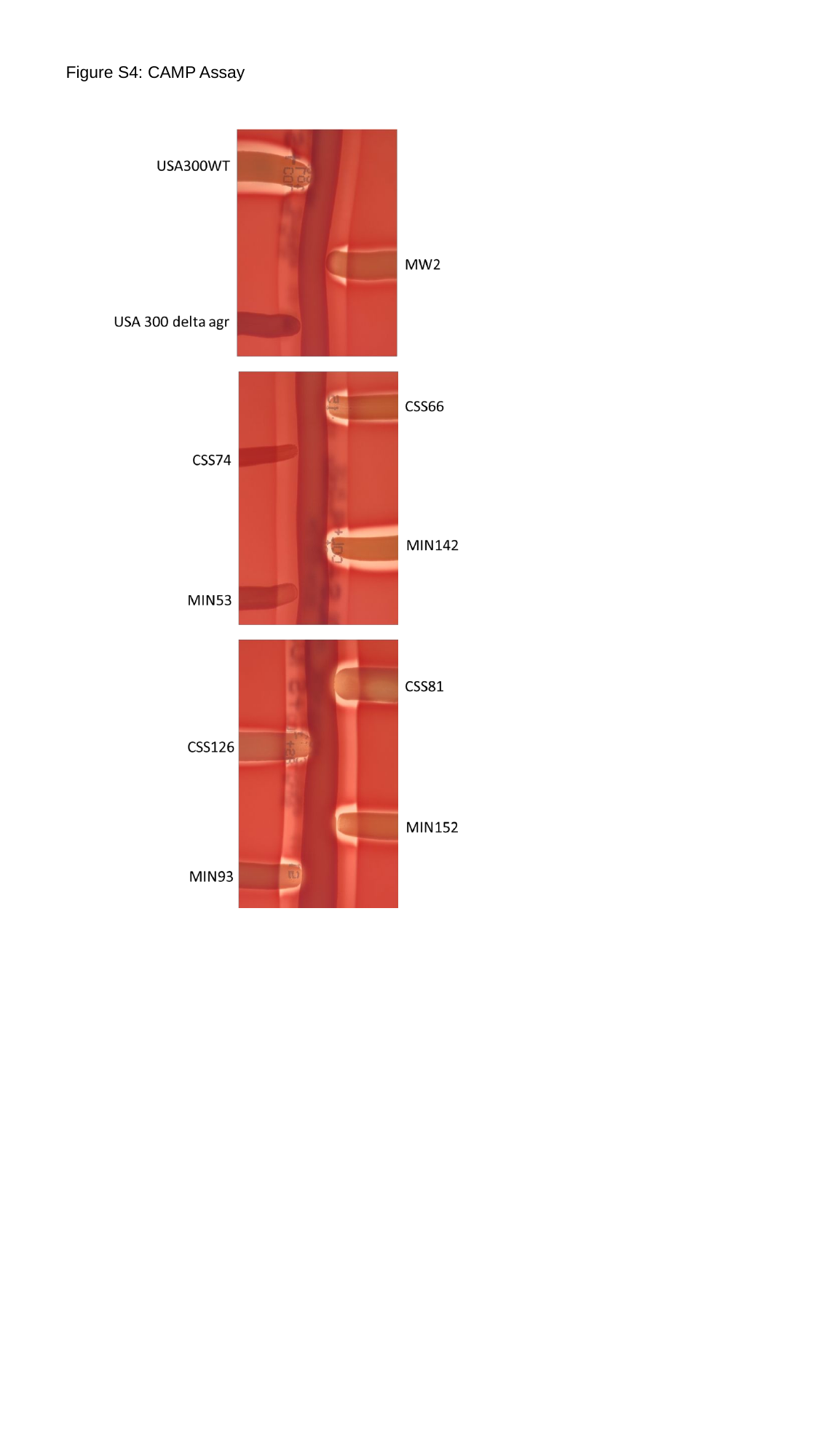

Figure S4: CAMP Assay

## Slide 6
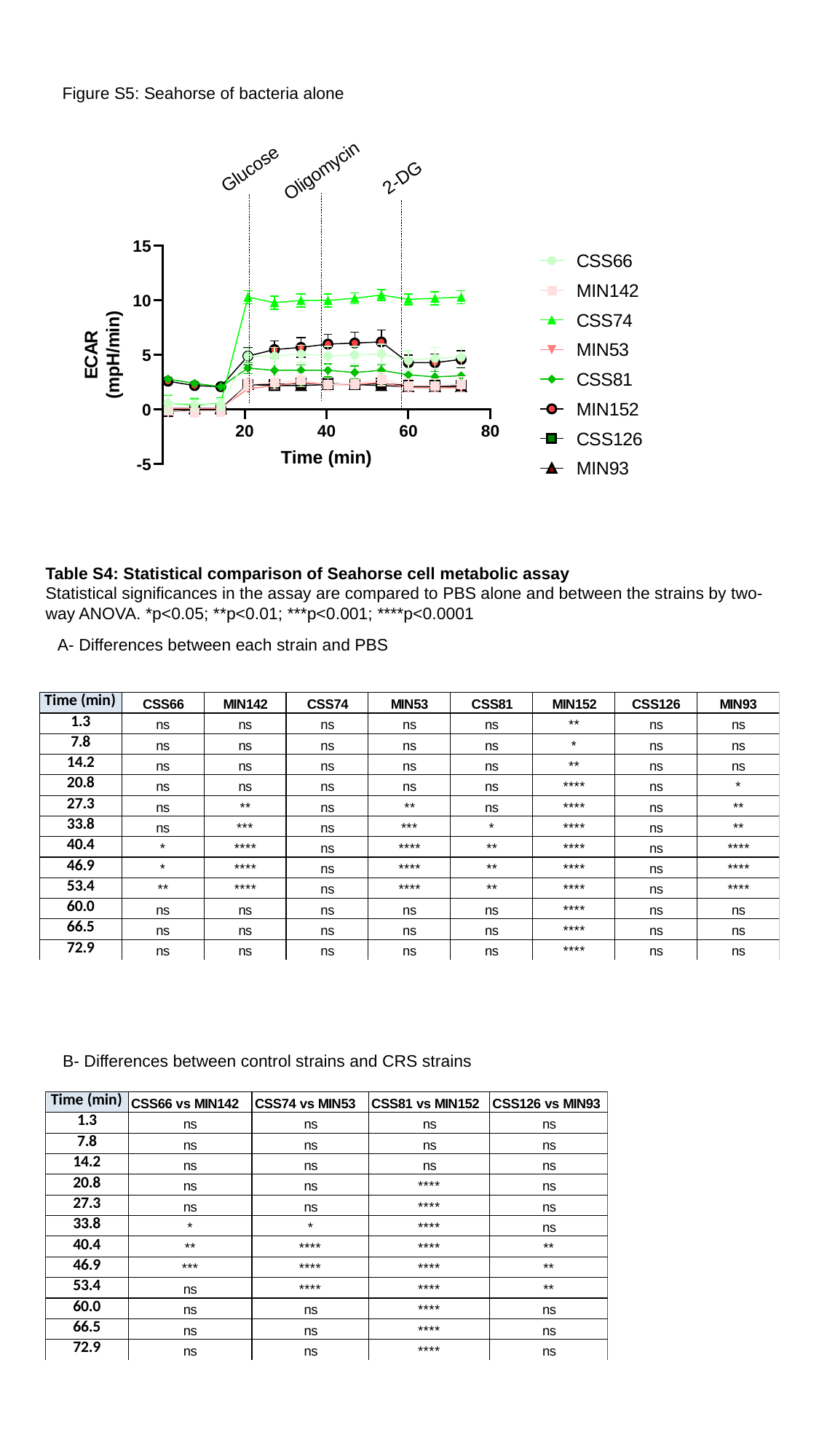

Figure S5: Seahorse of bacteria alone
Table S4: Statistical comparison of Seahorse cell metabolic assay
Statistical significances in the assay are compared to PBS alone and between the strains by two-way ANOVA. *p<0.05; **p<0.01; ***p<0.001; ****p<0.0001
A- Differences between each strain and PBS
B- Differences between control strains and CRS strains
